# Bis(*N*-nitroso-*N*-phenyl­hydroxy­laminato-κ^2^ *O*,*O*′)(1,10-phenanthroline-κ^2^ *N*,*N*′)lead(II)

**DOI:** 10.1107/S1600536811006787

**Published:** 2011-02-26

**Authors:** Ezzatollah Najafi, Mostafa M. Amini, Seik Weng Ng

**Affiliations:** a Department of Chemistry, General Campus, Shahid Beheshti University, Tehran 1983963113, Iran; b Department of Chemistry, University of Malaya, 50603 Kuala Lumpur, Malaysia

## Abstract

The two cupferronate ions and the *N*-heterocycle in the mononuclear title compound, [Pb(C_6_H_5_N_2_O_2_)_2_(C_12_H_8_N_2_)],  *O,O*′- and *N,N*′-chelate to the Pb^II^ atom, the geometry of which is a distorted Ψ-penta­gonal bipyramid.

## Related literature

For the structure of dinuclear [Pb(C_6_H_5_N_2_O_2_)_2_]_2_, see: Najafi *et al.* (2011 ▶).
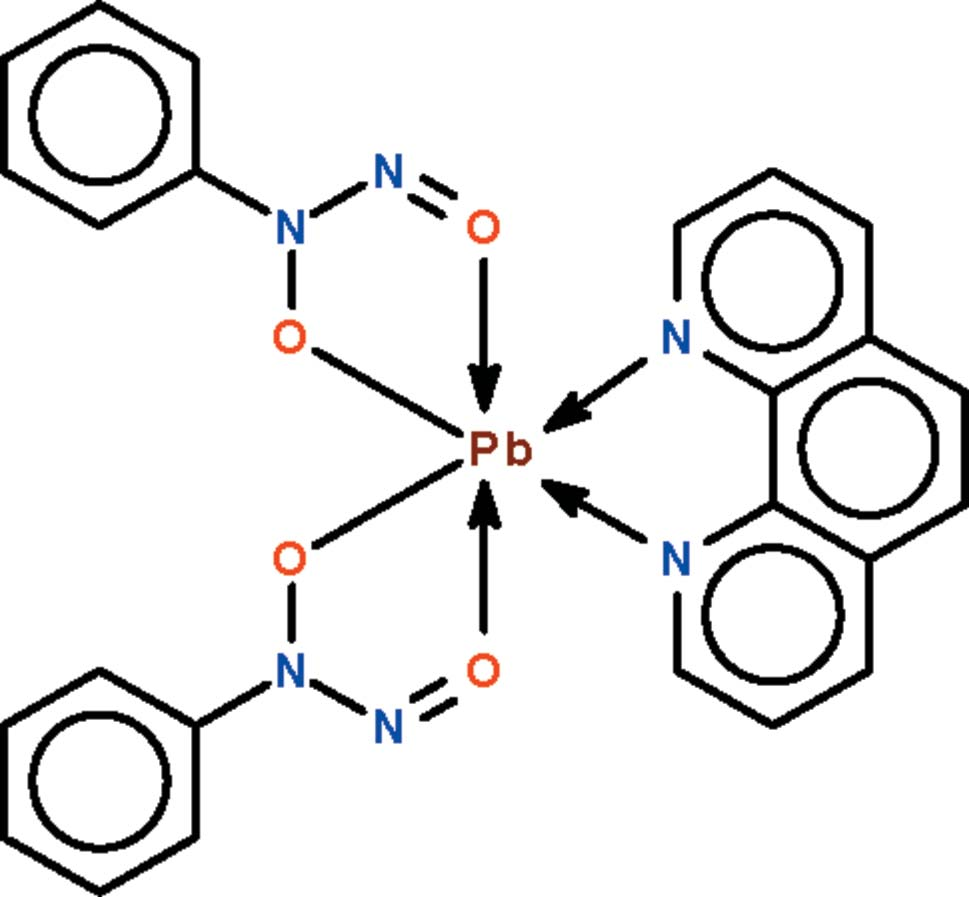


## Experimental

### 

#### Crystal data

[Pb(C_6_H_5_N_2_O_2_)_2_(C_12_H_8_N_2_)]
*M* *_r_* = 661.63
Monoclinic, 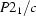

*a* = 7.7033 (4) Å
*b* = 15.9948 (8) Å
*c* = 18.8929 (10) Å
β = 100.919 (1)°
*V* = 2285.7 (2) Å^3^
*Z* = 4
Mo *K*α radiation
μ = 7.43 mm^−1^
*T* = 100 K
0.20 × 0.10 × 0.10 mm

#### Data collection

Bruker SMART APEX diffractometer
Absorption correction: multi-scan (*SADABS*; Sheldrick, 1996 ▶) *T*_min_ = 0.318, *T*_max_ = 0.524
21418 measured reflections
5233 independent reflections
4676 reflections with *I* > 2σ(*I*)
*R*_int_ = 0.028

#### Refinement

*R*[*F* ^2^ > 2σ(*F* ^2^)] = 0.018
*wR*(*F* ^2^) = 0.047
*S* = 0.91
5233 reflections
316 parameters
H-atom parameters constrained
Δρ_max_ = 0.74 e Å^−3^
Δρ_min_ = −0.53 e Å^−3^

### 

Data collection: *APEX2* (Bruker, 2009 ▶); cell refinement: *SAINT* (Bruker, 2009 ▶); data reduction: *SAINT*; program(s) used to solve structure: *SHELXS97* (Sheldrick, 2008 ▶); program(s) used to refine structure: *SHELXL97* (Sheldrick, 2008 ▶); molecular graphics: *X-SEED* (Barbour, 2001 ▶); software used to prepare material for publication: *publCIF* (Westrip, 2010 ▶).

## Supplementary Material

Crystal structure: contains datablocks global, I. DOI: 10.1107/S1600536811006787/bt5481sup1.cif

Structure factors: contains datablocks I. DOI: 10.1107/S1600536811006787/bt5481Isup2.hkl

Additional supplementary materials:  crystallographic information; 3D view; checkCIF report

## Figures and Tables

**Fig. 1.**
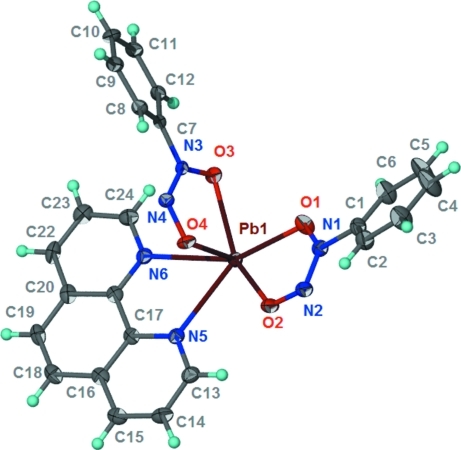
Anisotropic displacement ellipsoid plot (Barbour, 2001) of Pb(C12H8N2)(C6H5N2O2)2 at the 70% probability level. Hydrogen atoms are drawn as spheres of arbitrary radius.

## supplementary crystallographic information

### Comment

The cupferronate ion is a common ion used for the complexation of metals; the lead(II) derivative exists as a dinuclear compound, the four cupferronate ions in dinuclear [Pb(C_6_H_5_N_2_O_2_)_2_]_2_*O*,*O*'-chelate to the lead(II) atom, and two of the four nitroso O atoms are also involved in bridging. The geometry of both five-coordinate lead atoms is Ψ-octahedral; if another longer intermolecular Pb···O interactions (approx. 3.0 Å) are considered, the geometry is a Ψ-square-antiprism (Najafi *et al.*, 2011). The 1,10-phenanthroline adduct is monomeric (Scheme I, Fig. 1). The two cupferronate ions and the *N*-heterocycle in mononuclear Pb(C_12_H_8_N_2_)(C_6_H_5_N_2_O_2_)_2_ chelate to the lead(II) atom; the geometry of the lead atom is a Ψ-pentagonal bipyramid.

### Experimental

Lead(II) nitrate (0.33 g, 1 mmol) dissolved in ethanol (20 ml) was added to the cupferron ligand (0.31 g, 2 mmol) and 1,10-phenanthroline hydrate (0.40, 2 mmol) dissolved in ethanol (20 ml). The mixture was stirred and then set aside for the growth of brown colored crystals.

### Refinement

Hydrogen atoms were placed in calculated positions (C–H 0.95 Å) and were included in the refinement in the riding model approximation, with *U*(H) set to 1.2*U*_eq_(C).

Omitted from the refinement were the following reflections owing to bad disagreement between the observed and calculated *F*^2^ values: (0 0 1), (0 1 2), (1 0 1), (0 0 2), (11 4 7), (-9 - 11 5), (11 3 8), (11 5 6), (-4 - 9 10), (-9 - 9 2) and (3 - 2 14).

### Crystal data

**Table:**

[Pb(C_6_H_5_N_2_O_2_)_2_(C_12_H_8_N_2_)]	*F*(000) = 1272
*M**_r_* = 661.63	*D*_x_ = 1.923 Mg m^−^^3^
Monoclinic, *P*2_1_/*c*	Mo *K*α radiation, λ = 0.71073 Å
Hall symbol: -P 2ybc	Cell parameters from 9896 reflections
*a* = 7.7033 (4) Å	θ = 2.2–28.3°
*b* = 15.9948 (8) Å	µ = 7.43 mm^−^^1^
*c* = 18.8929 (10) Å	*T* = 100 K
β = 100.919 (1)°	Prism, brown
*V* = 2285.7 (2) Å^3^	0.20 × 0.10 × 0.10 mm
*Z* = 4	

### Data collection

**Table:**

Bruker SMART APEX diffractometer	5233 independent reflections
Radiation source: fine-focus sealed tube	4676 reflections with *I* > 2σ(*I*)
graphite	*R*_int_ = 0.028
ω scans	θ_max_ = 27.5°, θ_min_ = 2.2°
Absorption correction: multi-scan (*SADABS*; Sheldrick, 1996)	*h* = −9→10
*T*_min_ = 0.318, *T*_max_ = 0.524	*k* = −20→18
21418 measured reflections	*l* = −24→24

### Refinement

**Table:**

Refinement on *F*^2^	Primary atom site location: structure-invariant direct methods
Least-squares matrix: full	Secondary atom site location: difference Fourier map
*R*[*F*^2^ > 2σ(*F*^2^)] = 0.018	Hydrogen site location: inferred from neighbouring sites
*wR*(*F*^2^) = 0.047	H-atom parameters constrained
*S* = 0.91	*w* = 1/[σ^2^(*F*_o_^2^) + (0.0315*P*)^2^ + 1.0382*P*] where *P* = (*F*_o_^2^ + 2*F*_c_^2^)/3
5233 reflections	(Δ/σ)_max_ = 0.001
316 parameters	Δρ_max_ = 0.74 e Å^−^^3^
0 restraints	Δρ_min_ = −0.53 e Å^−^^3^

### Fractional atomic coordinates and isotropic or equivalent isotropic displacement parameters (Å^2^)

**Table:**

	*x*	*y*	*z*	*U*_iso_*/*U*_eq_	
Pb1	0.545412 (12)	0.564925 (6)	0.707994 (5)	0.01407 (4)	
O1	0.6518 (3)	0.57008 (11)	0.83910 (10)	0.0215 (4)	
O2	0.6004 (3)	0.43217 (11)	0.77043 (10)	0.0221 (4)	
O3	0.3666 (2)	0.67625 (11)	0.74664 (10)	0.0171 (4)	
O4	0.2682 (2)	0.52341 (11)	0.72411 (10)	0.0168 (4)	
N1	0.6651 (3)	0.49786 (14)	0.87218 (11)	0.0167 (4)	
N2	0.6420 (3)	0.42654 (14)	0.84043 (12)	0.0207 (5)	
N3	0.2048 (3)	0.65180 (13)	0.74731 (10)	0.0123 (4)	
N4	0.1479 (3)	0.57674 (12)	0.73517 (11)	0.0139 (4)	
N5	0.4379 (3)	0.44824 (13)	0.60458 (12)	0.0156 (4)	
N6	0.3223 (3)	0.61102 (14)	0.58176 (11)	0.0160 (4)	
C1	0.7119 (4)	0.49851 (17)	0.94969 (14)	0.0189 (5)	
C2	0.6692 (4)	0.43069 (17)	0.98924 (15)	0.0228 (6)	
H2	0.6111	0.3831	0.9657	0.027*	
C3	0.7135 (5)	0.43430 (18)	1.06373 (16)	0.0298 (7)	
H3	0.6877	0.3882	1.0917	0.036*	
C4	0.7955 (5)	0.5050 (2)	1.09786 (15)	0.0355 (8)	
H4	0.8229	0.5075	1.1490	0.043*	
C5	0.8371 (5)	0.57181 (19)	1.05735 (16)	0.0345 (8)	
H5	0.8947	0.6196	1.0808	0.041*	
C6	0.7950 (4)	0.56902 (18)	0.98277 (15)	0.0254 (6)	
H6	0.8225	0.6147	0.9548	0.031*	
C7	0.0758 (3)	0.71352 (15)	0.75744 (13)	0.0132 (5)	
C8	−0.0737 (3)	0.68857 (16)	0.78329 (13)	0.0159 (5)	
H8	−0.0865	0.6325	0.7980	0.019*	
C9	−0.2042 (4)	0.74779 (17)	0.78711 (14)	0.0193 (5)	
H9	−0.3089	0.7319	0.8034	0.023*	
C10	−0.1817 (4)	0.83009 (17)	0.76720 (14)	0.0211 (6)	
H10	−0.2722	0.8701	0.7686	0.025*	
C11	−0.0270 (4)	0.85391 (16)	0.74523 (14)	0.0187 (5)	
H11	−0.0099	0.9108	0.7338	0.022*	
C12	0.1030 (3)	0.79543 (15)	0.73976 (13)	0.0156 (5)	
H12	0.2084	0.8115	0.7242	0.019*	
C13	0.4890 (4)	0.36929 (17)	0.61474 (14)	0.0195 (6)	
H13	0.5640	0.3550	0.6590	0.023*	
C14	0.4390 (4)	0.30593 (17)	0.56419 (14)	0.0213 (6)	
H14	0.4786	0.2502	0.5743	0.026*	
C15	0.3320 (4)	0.32538 (17)	0.49995 (14)	0.0202 (6)	
H15	0.2974	0.2832	0.4647	0.024*	
C16	0.2737 (3)	0.40803 (17)	0.48634 (14)	0.0174 (5)	
C17	0.3300 (3)	0.46863 (17)	0.54094 (13)	0.0153 (5)	
C18	0.1582 (4)	0.43202 (17)	0.42116 (14)	0.0204 (6)	
H18	0.1205	0.3912	0.3850	0.024*	
C19	0.1015 (4)	0.51195 (18)	0.41007 (14)	0.0198 (6)	
H19	0.0243	0.5263	0.3664	0.024*	
C20	0.1563 (4)	0.57524 (16)	0.46329 (14)	0.0177 (5)	
C21	0.2698 (3)	0.55380 (16)	0.52921 (13)	0.0156 (5)	
C22	0.0991 (4)	0.65833 (18)	0.45407 (14)	0.0210 (6)	
H22	0.0237	0.6750	0.4106	0.025*	
C23	0.1520 (4)	0.71536 (18)	0.50770 (14)	0.0217 (6)	
H23	0.1136	0.7718	0.5021	0.026*	
C24	0.2643 (3)	0.68889 (17)	0.57130 (14)	0.0194 (5)	
H24	0.3002	0.7287	0.6085	0.023*	

### Atomic displacement parameters (Å^2^)

**Table:**

	*U*^11^	*U*^22^	*U*^33^	*U*^12^	*U*^13^	*U*^23^
Pb1	0.01178 (6)	0.01599 (6)	0.01461 (5)	−0.00027 (4)	0.00298 (4)	−0.00125 (3)
O1	0.0316 (12)	0.0155 (10)	0.0164 (9)	−0.0056 (8)	0.0021 (8)	0.0018 (7)
O2	0.0287 (12)	0.0188 (10)	0.0172 (9)	0.0091 (8)	0.0003 (8)	−0.0034 (7)
O3	0.0097 (8)	0.0152 (9)	0.0267 (9)	−0.0040 (7)	0.0043 (7)	−0.0032 (7)
O4	0.0155 (9)	0.0112 (9)	0.0250 (9)	0.0015 (7)	0.0073 (7)	−0.0010 (7)
N1	0.0163 (11)	0.0166 (11)	0.0173 (10)	0.0003 (9)	0.0031 (8)	0.0008 (9)
N2	0.0243 (13)	0.0184 (12)	0.0181 (11)	0.0058 (9)	0.0008 (9)	−0.0022 (9)
N3	0.0109 (10)	0.0117 (10)	0.0137 (9)	0.0006 (8)	0.0006 (8)	0.0003 (8)
N4	0.0140 (11)	0.0102 (10)	0.0175 (10)	−0.0011 (8)	0.0033 (8)	0.0001 (8)
N5	0.0143 (11)	0.0172 (11)	0.0162 (10)	0.0009 (9)	0.0049 (9)	0.0002 (8)
N6	0.0139 (11)	0.0187 (11)	0.0160 (10)	0.0012 (9)	0.0047 (8)	0.0001 (9)
C1	0.0192 (14)	0.0211 (14)	0.0157 (12)	0.0002 (11)	0.0015 (10)	0.0006 (10)
C2	0.0258 (16)	0.0186 (14)	0.0225 (14)	−0.0020 (11)	0.0004 (11)	0.0013 (11)
C3	0.043 (2)	0.0256 (16)	0.0197 (14)	−0.0043 (14)	0.0019 (13)	0.0061 (11)
C4	0.058 (2)	0.0329 (18)	0.0131 (13)	−0.0125 (16)	−0.0011 (13)	0.0020 (12)
C5	0.053 (2)	0.0283 (17)	0.0179 (14)	−0.0126 (15)	−0.0028 (14)	−0.0020 (12)
C6	0.0319 (17)	0.0239 (16)	0.0197 (13)	−0.0081 (12)	0.0031 (12)	0.0041 (11)
C7	0.0126 (12)	0.0131 (12)	0.0128 (11)	0.0004 (9)	−0.0002 (9)	−0.0021 (9)
C8	0.0152 (13)	0.0117 (12)	0.0204 (12)	−0.0026 (10)	0.0025 (10)	−0.0019 (10)
C9	0.0158 (13)	0.0186 (14)	0.0237 (13)	−0.0008 (11)	0.0041 (11)	−0.0066 (10)
C10	0.0202 (14)	0.0175 (13)	0.0249 (13)	0.0068 (11)	0.0023 (11)	−0.0047 (11)
C11	0.0245 (15)	0.0108 (12)	0.0193 (12)	0.0002 (11)	0.0007 (11)	−0.0009 (10)
C12	0.0167 (13)	0.0130 (12)	0.0163 (11)	−0.0010 (10)	0.0010 (10)	−0.0005 (10)
C13	0.0178 (14)	0.0215 (14)	0.0202 (13)	0.0035 (11)	0.0059 (11)	−0.0002 (11)
C14	0.0225 (14)	0.0148 (13)	0.0288 (14)	0.0008 (11)	0.0100 (11)	−0.0006 (11)
C15	0.0197 (14)	0.0196 (14)	0.0230 (13)	−0.0040 (11)	0.0082 (11)	−0.0043 (11)
C16	0.0140 (13)	0.0218 (13)	0.0186 (12)	−0.0045 (11)	0.0085 (10)	−0.0022 (10)
C17	0.0101 (12)	0.0196 (13)	0.0175 (12)	−0.0046 (10)	0.0059 (10)	−0.0019 (10)
C18	0.0192 (14)	0.0249 (15)	0.0180 (12)	−0.0082 (11)	0.0060 (11)	−0.0036 (11)
C19	0.0163 (13)	0.0294 (15)	0.0135 (12)	−0.0051 (11)	0.0020 (10)	0.0003 (10)
C20	0.0145 (13)	0.0254 (15)	0.0146 (12)	−0.0024 (11)	0.0062 (10)	0.0024 (10)
C21	0.0127 (13)	0.0206 (14)	0.0152 (12)	−0.0025 (10)	0.0068 (10)	−0.0008 (10)
C22	0.0143 (13)	0.0293 (15)	0.0188 (12)	0.0008 (11)	0.0018 (10)	0.0078 (11)
C23	0.0207 (14)	0.0219 (15)	0.0238 (13)	0.0004 (11)	0.0073 (11)	0.0044 (11)
C24	0.0198 (13)	0.0187 (13)	0.0212 (12)	0.0003 (11)	0.0080 (10)	−0.0002 (10)

### Geometric parameters (Å, °)

**Table:**

Pb1—O4	2.3106 (18)	C7—C8	1.392 (3)
Pb1—O2	2.4270 (18)	C8—C9	1.393 (4)
Pb1—O3	2.4457 (17)	C8—H8	0.9500
Pb1—O1	2.4586 (19)	C9—C10	1.389 (4)
Pb1—N5	2.715 (2)	C9—H9	0.9500
Pb1—N6	2.763 (2)	C10—C11	1.387 (4)
O1—N1	1.308 (3)	C10—H10	0.9500
O2—N2	1.304 (3)	C11—C12	1.389 (4)
O3—N3	1.309 (3)	C11—H11	0.9500
O4—N4	1.305 (3)	C12—H12	0.9500
N1—N2	1.285 (3)	C13—C14	1.396 (4)
N1—C1	1.440 (3)	C13—H13	0.9500
N3—N4	1.284 (3)	C14—C15	1.367 (4)
N3—C7	1.439 (3)	C14—H14	0.9500
N5—C13	1.326 (3)	C15—C16	1.404 (4)
N5—C17	1.365 (3)	C15—H15	0.9500
N6—C24	1.325 (3)	C16—C17	1.422 (4)
N6—C21	1.354 (3)	C16—C18	1.428 (4)
C1—C6	1.386 (4)	C17—C21	1.443 (4)
C1—C2	1.392 (4)	C18—C19	1.354 (4)
C2—C3	1.385 (4)	C18—H18	0.9500
C2—H2	0.9500	C19—C20	1.433 (4)
C3—C4	1.392 (4)	C19—H19	0.9500
C3—H3	0.9500	C20—C22	1.401 (4)
C4—C5	1.387 (4)	C20—C21	1.421 (4)
C4—H4	0.9500	C22—C23	1.367 (4)
C5—C6	1.385 (4)	C22—H22	0.9500
C5—H5	0.9500	C23—C24	1.406 (4)
C6—H6	0.9500	C23—H23	0.9500
C7—C12	1.378 (3)	C24—H24	0.9500
			
O4—Pb1—O2	76.42 (7)	C8—C7—N3	119.2 (2)
O4—Pb1—O3	65.37 (6)	C7—C8—C9	118.5 (2)
O2—Pb1—O3	123.25 (6)	C7—C8—H8	120.8
O4—Pb1—O1	90.97 (7)	C9—C8—H8	120.8
O2—Pb1—O1	62.97 (6)	C10—C9—C8	120.1 (2)
O3—Pb1—O1	76.93 (6)	C10—C9—H9	119.9
O4—Pb1—N5	74.56 (6)	C8—C9—H9	119.9
O2—Pb1—N5	75.51 (6)	C11—C10—C9	120.0 (2)
O3—Pb1—N5	127.08 (6)	C11—C10—H10	120.0
O1—Pb1—N5	138.25 (6)	C9—C10—H10	120.0
O4—Pb1—N6	75.59 (6)	C10—C11—C12	120.7 (2)
O2—Pb1—N6	132.52 (7)	C10—C11—H11	119.7
O3—Pb1—N6	76.70 (6)	C12—C11—H11	119.7
O1—Pb1—N6	153.50 (7)	C7—C12—C11	118.5 (2)
N5—Pb1—N6	60.47 (6)	C7—C12—H12	120.8
N1—O1—Pb1	115.64 (14)	C11—C12—H12	120.8
N2—O2—Pb1	122.70 (14)	N5—C13—C14	123.8 (3)
N3—O3—Pb1	112.12 (13)	N5—C13—H13	118.1
N4—O4—Pb1	122.37 (14)	C14—C13—H13	118.1
N2—N1—O1	124.7 (2)	C15—C14—C13	118.9 (3)
N2—N1—C1	117.8 (2)	C15—C14—H14	120.5
O1—N1—C1	117.5 (2)	C13—C14—H14	120.5
N1—N2—O2	113.4 (2)	C14—C15—C16	119.7 (2)
N4—N3—O3	124.9 (2)	C14—C15—H15	120.2
N4—N3—C7	116.4 (2)	C16—C15—H15	120.2
O3—N3—C7	118.64 (19)	C15—C16—C17	117.8 (2)
N3—N4—O4	114.2 (2)	C15—C16—C18	122.4 (2)
C13—N5—C17	118.0 (2)	C17—C16—C18	119.8 (3)
C13—N5—Pb1	120.57 (17)	N5—C17—C16	121.8 (2)
C17—N5—Pb1	121.42 (16)	N5—C17—C21	119.0 (2)
C24—N6—C21	118.7 (2)	C16—C17—C21	119.2 (2)
C24—N6—Pb1	121.10 (17)	C19—C18—C16	121.1 (2)
C21—N6—Pb1	120.19 (16)	C19—C18—H18	119.4
C6—C1—C2	121.9 (2)	C16—C18—H18	119.4
C6—C1—N1	118.0 (2)	C18—C19—C20	121.0 (2)
C2—C1—N1	120.1 (2)	C18—C19—H19	119.5
C3—C2—C1	118.4 (3)	C20—C19—H19	119.5
C3—C2—H2	120.8	C22—C20—C21	117.7 (2)
C1—C2—H2	120.8	C22—C20—C19	122.6 (2)
C2—C3—C4	120.5 (3)	C21—C20—C19	119.7 (2)
C2—C3—H3	119.8	N6—C21—C20	121.9 (2)
C4—C3—H3	119.8	N6—C21—C17	118.9 (2)
C5—C4—C3	120.2 (3)	C20—C21—C17	119.3 (2)
C5—C4—H4	119.9	C23—C22—C20	119.9 (3)
C3—C4—H4	119.9	C23—C22—H22	120.0
C6—C5—C4	120.1 (3)	C20—C22—H22	120.0
C6—C5—H5	119.9	C22—C23—C24	118.8 (3)
C4—C5—H5	119.9	C22—C23—H23	120.6
C1—C6—C5	119.0 (3)	C24—C23—H23	120.6
C1—C6—H6	120.5	N6—C24—C23	123.1 (3)
C5—C6—H6	120.5	N6—C24—H24	118.5
C12—C7—C8	122.1 (2)	C23—C24—H24	118.5
C12—C7—N3	118.6 (2)		
			
O4—Pb1—O1—N1	68.29 (18)	C1—C2—C3—C4	1.3 (5)
O2—Pb1—O1—N1	−5.91 (16)	C2—C3—C4—C5	−1.4 (6)
O3—Pb1—O1—N1	132.71 (18)	C3—C4—C5—C6	1.0 (6)
N5—Pb1—O1—N1	0.7 (2)	C2—C1—C6—C5	0.3 (5)
N6—Pb1—O1—N1	126.70 (18)	N1—C1—C6—C5	178.4 (3)
O4—Pb1—O2—N2	−92.0 (2)	C4—C5—C6—C1	−0.4 (5)
O3—Pb1—O2—N2	−44.2 (2)	N4—N3—C7—C12	−152.2 (2)
O1—Pb1—O2—N2	6.17 (19)	O3—N3—C7—C12	23.7 (3)
N5—Pb1—O2—N2	−169.3 (2)	N4—N3—C7—C8	26.5 (3)
N6—Pb1—O2—N2	−147.37 (18)	O3—N3—C7—C8	−157.6 (2)
O4—Pb1—O3—N3	−7.02 (13)	C12—C7—C8—C9	3.9 (4)
O2—Pb1—O3—N3	−59.47 (16)	N3—C7—C8—C9	−174.7 (2)
O1—Pb1—O3—N3	−104.23 (15)	C7—C8—C9—C10	−1.7 (4)
N5—Pb1—O3—N3	37.44 (17)	C8—C9—C10—C11	−1.7 (4)
N6—Pb1—O3—N3	73.02 (14)	C9—C10—C11—C12	2.9 (4)
O2—Pb1—O4—N4	146.36 (18)	C8—C7—C12—C11	−2.7 (4)
O3—Pb1—O4—N4	9.37 (16)	N3—C7—C12—C11	176.0 (2)
O1—Pb1—O4—N4	84.50 (17)	C10—C11—C12—C7	−0.8 (4)
N5—Pb1—O4—N4	−135.20 (18)	C17—N5—C13—C14	0.0 (4)
N6—Pb1—O4—N4	−72.39 (17)	Pb1—N5—C13—C14	179.90 (19)
Pb1—O1—N1—N2	6.5 (3)	N5—C13—C14—C15	0.5 (4)
Pb1—O1—N1—C1	−174.66 (17)	C13—C14—C15—C16	−0.5 (4)
O1—N1—N2—O2	−1.0 (4)	C14—C15—C16—C17	0.2 (4)
C1—N1—N2—O2	−179.9 (2)	C14—C15—C16—C18	−178.3 (2)
Pb1—O2—N2—N1	−5.4 (3)	C13—N5—C17—C16	−0.4 (4)
Pb1—O3—N3—N4	5.5 (3)	Pb1—N5—C17—C16	179.72 (17)
Pb1—O3—N3—C7	−170.04 (15)	C13—N5—C17—C21	178.8 (2)
O3—N3—N4—O4	2.4 (3)	Pb1—N5—C17—C21	−1.1 (3)
C7—N3—N4—O4	177.98 (19)	C15—C16—C17—N5	0.3 (4)
Pb1—O4—N4—N3	−10.2 (3)	C18—C16—C17—N5	178.9 (2)
O4—Pb1—N5—C13	−96.9 (2)	C15—C16—C17—C21	−178.9 (2)
O2—Pb1—N5—C13	−17.25 (19)	C18—C16—C17—C21	−0.3 (4)
O3—Pb1—N5—C13	−138.21 (18)	C15—C16—C18—C19	178.8 (3)
O1—Pb1—N5—C13	−23.3 (2)	C17—C16—C18—C19	0.3 (4)
N6—Pb1—N5—C13	−178.8 (2)	C16—C18—C19—C20	0.3 (4)
O4—Pb1—N5—C17	83.03 (18)	C18—C19—C20—C22	−179.3 (3)
O2—Pb1—N5—C17	162.6 (2)	C18—C19—C20—C21	−0.9 (4)
O3—Pb1—N5—C17	41.7 (2)	C24—N6—C21—C20	0.6 (4)
O1—Pb1—N5—C17	156.59 (17)	Pb1—N6—C21—C20	−179.48 (18)
N6—Pb1—N5—C17	1.10 (17)	C24—N6—C21—C17	−178.8 (2)
O4—Pb1—N6—C24	98.62 (19)	Pb1—N6—C21—C17	1.0 (3)
O2—Pb1—N6—C24	154.25 (17)	C22—C20—C21—N6	−0.1 (4)
O3—Pb1—N6—C24	31.04 (18)	C19—C20—C21—N6	−178.6 (2)
O1—Pb1—N6—C24	37.1 (3)	C22—C20—C21—C17	179.4 (2)
N5—Pb1—N6—C24	178.8 (2)	C19—C20—C21—C17	0.9 (4)
O4—Pb1—N6—C21	−81.27 (18)	N5—C17—C21—N6	0.0 (3)
O2—Pb1—N6—C21	−25.6 (2)	C16—C17—C21—N6	179.2 (2)
O3—Pb1—N6—C21	−148.84 (19)	N5—C17—C21—C20	−179.5 (2)
O1—Pb1—N6—C21	−142.83 (17)	C16—C17—C21—C20	−0.3 (4)
N5—Pb1—N6—C21	−1.08 (17)	C21—C20—C22—C23	−0.4 (4)
N2—N1—C1—C6	158.2 (3)	C19—C20—C22—C23	178.1 (2)
O1—N1—C1—C6	−20.8 (4)	C20—C22—C23—C24	0.3 (4)
N2—N1—C1—C2	−23.7 (4)	C21—N6—C24—C23	−0.7 (4)
O1—N1—C1—C2	157.4 (3)	Pb1—N6—C24—C23	179.37 (18)
C6—C1—C2—C3	−0.7 (5)	C22—C23—C24—N6	0.3 (4)
N1—C1—C2—C3	−178.8 (3)		
